# Breed-specific gut microbiota and enterotype divergence in Chinese indigenous ducks

**DOI:** 10.3389/fmicb.2025.1602641

**Published:** 2025-07-17

**Authors:** Yongfei Wu, Jing Ouyang, Luping Wang, Jingyan Hu, Hongbo Tang, Sumei Zheng, Yanpeng Xiong, Yuren Gao, Yan Wu, Rui Xiong, Yuxuan Huang, Rui Xuan, Yanhua Chen, Hao Chen

**Affiliations:** Key Laboratory of Natural Microbial Medicine Research of Jiangxi Province, College of Life Sciences, Jiangxi Science and Technology Normal University, Nanchang, China

**Keywords:** host species, domestic duck, gut microbiota, 16S rRNA gene, microbial diversity

## Abstract

The gut microbiota of domestic ducks plays an important role in digestion and absorption, immune regulation, and overall health. However, our knowledge about the gut microbial composition in ducks of various phylogeny is insufficient, especially if raised in the same farm environment. In this study, 260 fecal samples from 15 Chinese indigenous duck breeds living in a uniformed farm were collected and 16 S rRNA gene sequencing was performed. In addition, 202 blood samples from these ducks were used for whole-genome sequencing (WGS). The WGS results showed that the these domestic duck breeds exhibit breed-specific genetic characteristics. The gut microbiota of different native duck breeds exhibited great similarity at the phylum level with the most dominant phyla being *Firmicutes*, *Proteobacteria*, *Actinobacteria* and *Bacteroidetes*, while harboring distinct gut microbial communities at finer taxonomic levels. The host genetic-specific are associated with the microbial of these duck breeds. The prediction of metagenomic functions showed that the metabolism and function of the gut microbiomes among different duck breeds were more similar than that of their species composition. In addition, Principal coordinates analysis (PCoA) revealed that the gut microbiota of the 15 duck breeds could be divided into two distinct enterotype clusters based on Jensen–Shannon distance (JSD) dissimilarities, with representative breeds corresponding to layer ducks and dual-purpose ducks, respectively. There was no difference in richness index of the gut microbial composition and function between the two enterotypes, but the Shannon index values was significantly different. This study investigated the gut microbial structure and diversity among domestic duck populations with different genetic backgrounds, providing new insights into the relationship between host genetic variation and gut microbiota.

## Introduction

Domestic duck is an important agricultural animal in China. It is a valuable protein source for humans, and an important experimental bird model. China has the greatest number of different domestic duck breeds in the world, with 32 indigenous breeds, most of which are distributed along the middle and lower reaches of the Yangtze river and the coastal districts. The health and development of domestic ducks are closely related to microbiome in the gastrointestinal tract (Yang et al., 2020). These gut microbiota play a positive role in promoting digestion, maintaining immune homeostasis, and defending against pathogen invasion (Ocejo et al., 2019). Therefore, defining what constitutes healthy gut microbiota is essential, and help us design strategies to adjust its structure to maintain the health and improve the production performance of host.

The gut microbiome of animals is a vast and complex microbial ecosystem. The composition and ecological succession of the gut microbiome are shaped by several complex external and internal factors (Guevarra et al., 2019). Most animals acquire their microbes vertically from their parents or horizontally from food and environment throughout their lives (Maraci et al., 2021). The richness and diversity of fecal microbiota of domestic ducks showed significant variation in both spatial and temporal distribution. Zhu et al. (2020) found that among the dominant phyla in the duodenum, jejunum, and ileum of the Gaoyou duck, Chlorobi, Saccharibacteria and WCHBI_60, and Spirochaetae were detected only in the duodenum, jejunum, and ileum, respectively. In the cecal contents of Pekin ducks, Proteobacteria was dominant in the first 3 days after birth, and then Firmicutes began to increase and dominate. At the later stage of development, in addition to Proteobacteria and Firmicutes, Beijing ducks raised in a greenhouse also harbored large numbers of Bacteroidota (Best et al., 2016). As ducks mature, the composition of their gut microbiota tends to stabilize and maintain homeostasis.

Recently, increasing numbers of studies on the effects of various factors on the gut microbiota of ducks, including feed composition, additives, and living environment (Michel et al., 2018). In poultry, diet is one of the main factors affecting the gut microbial composition, including dietary ingredients, such as proteins, carbohydrates, and antibiotics/drugs (Rehman et al., 2007; Wang et al., 2022; Wu et al., 2020; Zheng et al., 2023). In addition, different captive environments, such as the traditional rearing condition and dryland rearing on netting floor (Zhao et al., 2019), the rearing floor type (Wang et al., 2018) and the temperature of the rearing environments (Tian et al., 2020) can cause significant differences in gut microbiota. However, even if the birds live in the same environment, there are still great differences in certain taxonomic levels among gut microbiota of different populations, which may be caused by different genetic backgrounds (Wang et al., 2019). Many studies have proven the genetic relationship between gut microbiota and host, including human (Deschasaux et al., 2018), chicken (Zhao et al., 2013), and tropical birds (Hird et al., 2015), but information on the effect of the genetic background on the gut microbial composition in ducks remains limited.

A model organism, kept in a stable environment with minimum environmental variations is required to unravel the effects of host genetics (Zhao et al., 2013). We collected fresh fecal samples from 15 local Chinese duck breeds and used 16S rRNA gene amplicon sequencing and whole genome resequencing to determine the composition of the gut microbiome of different breeds in the same environment and the genetic influence on the gut microbial composition as the ducks mature. While the 16S rRNA V4 region allows robust genus-level taxonomic assignment, its resolution for species-level discrimination is constrained. Thus, all biomarker and functional analyses were interpreted at the genus level. Our findings will improve our understanding of the gut microbiota in ducks and provide new insights into the association between gut microbiome and host genetics.

## Materials and methods

### Animal management and sample collection

In this study, a total of 260 fecal samples of 15 Chinese indigenous domestic duck breeds (120-days-old) and 202 blood samples of 13 groups were collected from the Waterfowl genetic Resources Conservation Research Center of Shishi City, Fujian Province (Supplementary Table S1). All experimental ducks were hatched on the same day and housed on the same breeding farm in similar living conditions, including appropriatest stocking density, diet, and rearing environment. These ducks had free access to a commercial formula feed primarily composed of corn and soybean meal. All experimental subjects remained healthy throughout the study and did not receive any antibiotic treatment. Blood samples were obtained via the wing veins and stored at −*20*°C until use; fecal samples was collected from the cloaca by squeezing the abdomen and stored at −*80*°C until use.

### DNA extraction, library preparation, and sequencing

Total genome DNA from the fecal samples was extracted using the CTAB extraction method, and the concentration and purity were monitored on 1% agarose gels. The 1 ng/μL genome DNA were used as templates for PCR reactions, and the V4 regions of 16 S rRNA genes were amplified using specific primers (forward primer 515 F and reverse primer 806 R) with barcode. An equal volume of 1 X loading buffer (containing SYB green) was mixed with the PCR product and electrophoresed on 2% agarose gel. The PCR products were mixed in equal density ratios and purified using the Qiagen Gel Extraction Kit (Qiagen, Germany). Sequencing libraries were obtained using TruSeq^®^ DNA PCR-Free Sample Preparation Kit (Illumina, United States) according to the manufacturer’s instructions and index codes were added. The quality of libraries was assessed using a Qubit@ 2.0 Fluorometer (Thermo Scientific) and Agilent Bioanalyzer 2,100 system. Finally, the libraries were sequenced using the Illumina NovaSeq6000 platform (Novogene, Beijing, China).

### Sequence data processing

According to their unique barcodes, the paired-end reads were assigned to samples and truncated by cutting off the barcode and primer sequences, and merged using FLASH (Magoc and Salzberg, 2011). The splicing sequences were called raw tags. Quality filtering was performed to obtain high-quality clean tags (Bokulich et al., 2013) according to the QIIME quality control process (Caporaso et al., 2010). These tags were compared with the reference database based on the UCHIME algorithm to detect and remove chimera sequences (Edgar et al., 2011; Haas et al., 2011), and effective tags were obtained. Sequences with ≥ 97% similarity were clustered into the same OTUs using Upaese software (Edgar, 2013), and the representative sequence of each OTU was screened for further annotation using the Silva Database (Quast et al., 2013). To obtain the phylogenetic relationship of different OTUs, multiple sequence alignment was performed using MUSCLE software (Edgar, 2004). OTU abundances were normalized using a standard of sequence number corresponding to the sample with the least sequences for further processing.

### Whole-genome sequencing and data processing

Duck DNA was extracted from the blood sample using a routine phenol/chloroform extraction protocol, and was commissioned from Novogene for whole-genome resequencing using Illumina NovaSeq platform. After resequencing, the raw data were filteres using the software SOAPnuke to obtain high-quality clean data. The clean reads were mapped against the duck reference genome using Burrows-Wheeler-Alignment Tool (BWA). The software SAMtools^1^ and GATK^2^ were used to detect variations and SNPs. The software of PLINK (v.1.90) was used for quality control with the filtering criteria: minor allele frequency (MAF) > 5%, missingness by genotype < 1%. In total, 8,993,736 SNPs were retained for subsequent analysis.

### Statistical analyses

The richness, Shannon, and PD whole tree indices values were calculated to evaluate the alpha diversities of the bacterial composition using the “vegan” package (version 2.5.7) in R. For beta diversity, we made principal coordinates analysis to investigate the dissimilarity of microbial communities using the Bray–Curtis distance. To determine the core microbiome in each duck breed, genus abundance > 0.1% was considered in at least one breed. The genus was defined as the core microbiome of the entire domestic duck population in more than 95% of individuals. To reveal the relationship between genera, we used the “psych” package (version 2.1.6) in R to calculate the pairwise Spearman’s rank correlation and adjusted the *p*-value to avoid false positives using the False discovery rate (FDR) method. Gephi software (version 0.9.2) was used to visualize the co-occurrence network. LEfSe analysis was performed to identify the breed-specific biomarkers at each taxonomic level. Microbial functions were predicted by Tax4Fun based on high-quality sequences.

### The influence of host genetics on the gut microbiota

To evaluated the population structure of the domestic duck, the principal component analysis (PCA) and the neighbor-joining (NJ) tree were conducted using PLINK (v.1.90). The Heterozygote rate and Homozygosity coefficient were calculated by PLINK (v1.90). The NJ tree was construct for individuals using iTOL (v.6.9.1). To explore the effects of the host genetics on the gut microbiota, we selected 202 domestic duck feces containing blood samples for follow-up study. 8,993,736 SNPs were used to estimate the genetic relatedness matrix (GRM) using GCTA (v1.93) and estimate the IBS matrix using PLINK (v1.90). And the correlation between the GRM and IBS matrix and Bray-Curtis distances was evaluated through Spearman’s correlation-based Mantel tests with 10,000 permutations, respectively.

### Application of enterotype clustering method

We applied methods described in humans to test for the presence of enterotypes in domestic ducks. The JSD and BC distances of the genera of samples were calculated in R, and the enterotype clustering of gut microbiotas was performed using the partitioning around medoid (PAM) method in the “cluster” package in R. The optimal number of clusters was selected according to the CH values that were calculated using the “clusterSim” package. The samples were plotted via principal coordinate analysis using the “ade4” package. SIMPER was applied to identify the genus taxa contributing to similarity within and dissimilarity between enterotypes and rank their contribution.

## Results

### The quality of sequencing data

An average of 63,545 effective sequences were recovered, and the average quality control response rate was 88.36% (Supplementary Table S2). A total of 14,722 operational taxonomic units (OTUs) with 97% sequence similarity threshold were identified in 15 duck breeds (Supplementary Table S3). We found that 688 bacterial OTUs representing 12 known phyla and 176 known genera were shared in each duck breed, which accounted for 76.71–96.32% of the duck gut microbiota (Supplementary Figure S1A). Only 19 core OTUs exist in each individual, while 39–741 OTUs were unique to each duck breed, but their abundances were low (0.014–0.582%). The sample size of domestic ducks collected in this study is large enough to cover most microorganisms in the intestine of domestic ducks (Supplementary Figure S1B). The rarefaction curves indicating that there were sufficient reads to represent each microbiome community (Supplementary Figure S1C). The rank abundance curve showed that a few dominant species accounted for a large proportion of gut microbiota in ducks (Supplementary Figure S1D).

**FIGURE 1:**
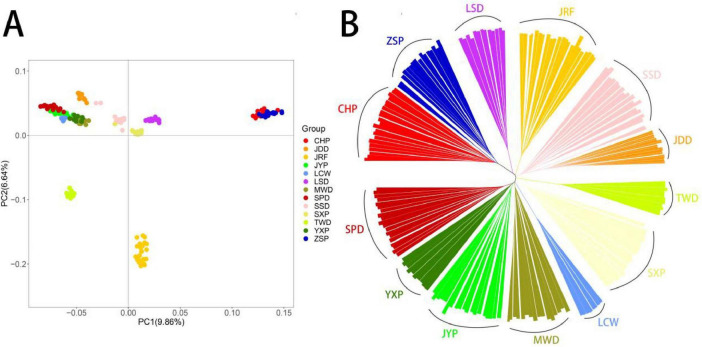
Genetic divergence among Chinese indigenous ducks. **(A)** PCA of variant data between the Chinese indigenous ducks based on the first and second principal components. **(B)** NJ-tree of Chinese indigenous ducks based on IBS distance.

To assess the genetic relationships among the 13 duck breeds, PCA, NJ tree analyses were conducted using 8,993,736 SNPs. The first principal component explained 9.86% of the total variation and was used to visually depict the genetic distance between CHP, ZSP and other breeds. The second principal component explained 6.64% of the total variance and was used to visually distinguish JRF, TWD and others (Figure 1A). The NJ tree showed clearly defined clusters, which the 13 duck breeds were separated into 13 distinct clusters, suggested that the 13 duck groups are genetically distinct breeds (Figure 1B).

### Gut microbial composition in different duck breeds

**FIGURE 2:**
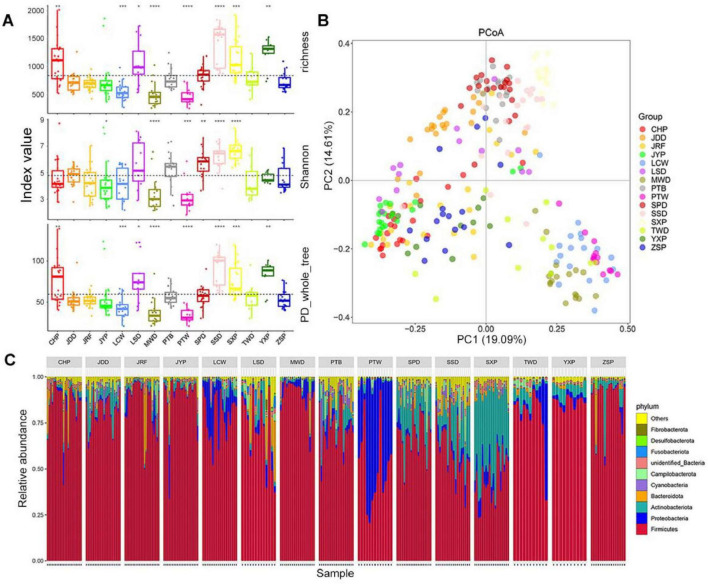
Comparison of gut microbiota composition among different duck breeds. **(A)** OTU alpha diversity. Boxes denote the interquartile (IQR) between the first and third quartiles (25th and 75th percentiles, respectively) and the line inside denotes the median. The asterisks on the top indicate *P < 0.05, **P < 0.01, ***P < 0.001, and ****P < 0.0001 (Wilcoxon test). **(B)** Principal coordinate analysis (PCoA) based on Bray–Curtis distance. **(C)** The relative abundance and distribution of the 10 most abundant phyla.

To minimize the disturbance of the lower abundant OTUs, the 5,133 OTUs with read numbers ≥ 3 in at least three samples were retained in this experiment. The results of alpha diversity based on OTU level showed that the richness and phylogenetic diversity (PD) whole tree indices values of gut microbiota in the Chaohu Partridge (CHP), Longsheng (LSD), Sansui (SSD), and Shaoxing Partridge Ducks (SXP) were significantly higher than the average (*P* < 0.05), and those of the Liancheng White (LCW), Mawang (MWD), and Putian White Ducks (PTW) were lower than the average (*P* < 0.05), and there was no significant difference in richness index values between other duck breeds and the average. The Wilcoxon test suggested that the Shannon index values in the Shan Partridge Duck (SPD), SSD, and SXP were significantly higher than the average, and those of Jinyun Partridge Duck (JYP), MWD, and PTW were significantly lower than the average (*P* < 0.05; Figure 2A). It is worth noting that these indices of gut microbiota were significantly higher in SSD and SXP, and lower in MWD and PTW.

For beta diversity, the results of the Principle Coordinates Analysis (PCoA) based on the Bray–Curtis distance revealed that gut microbiota from the same duck breed tended to gather together, and that of different breeds showed obvious segregation, in which the first principal component (PC1) and the second principal component (PC2) explained 19.09 and 14.61% of the differences in microbial structure, respectively (Figure 2B). The results of the unweighted pair-group method with arithmetic mean clustering tree (UPGMA-clustering tree) based on the Bray–Curtis distance was similar to that of the PCoA analysis; they verified each other, which reflected the clustering of the gut microbiota of each duck breed (Supplementary Figure S2A). The results of ANOSIM analysis showed that there were significant differences in gut microbiota among different duck breeds, which was greater than that within the same breed (*r* = 0.62, *P* < 0.001) (Supplementary Figure S2B).

At the phylum level, the gut microbiota of ducks was classified into 55 phyla. The most dominant phylum was Firmicutes, which accounted for 44.46–89.7% of total bacterial sequences from all duck breeds (Figure 2C; Supplementary Figure S2C). In addition, Proteobacteria was dominant in PTW (46.27%), LCW (14.87%) and the Taiwang Duck (TWD, 10.85%), and Actinobacteria was dominant in SXP (39.54%), SPD (19.03%), and SSD (17.47%). Bacteroidetes was dominant in LSD (8.63%), CHP (5.17%), and depleted in others (0.30–3.91%). Overall, Firmicutes, Actinobacteria, Proteobacteria, and Bacteroidetes accounted for 85.08–98.22% of the entire bacterial community, as the four dominant phyla in all duck breeds. At the genus level, 871 genera were identified across all samples, and the representative genera in each duck breed were diverse. Lactobacillus was the most common in most duck breeds, especially in JYP and CHP (the relative abundance > 50%, Supplementary Figure S2D). PTW had the uniquely enriched genus *Psychrobacter* (46%), whereas SXP harbored the most abundant genus *Brachybacterium* (15.91%). A large proportion of the genus, *Streptococcus*, was found in LCW (21.25%), MWD (47.77%), and TWD (30.27%). *Lactobacillus*, *Romboutsia*, and *Enterococcus* were common in the Putian Black Duck (PTB) with a relative abundance > 10%. However, except for *Enterococcus*, the proportion of these genera was low in PTW.

### The connection between host genetic and gut microbiota

To explore the influence of the host genetics on the gut microbial community, we verified the association between the GRM and microbial Bray-Curtis distance using mantel test, and found no correlation (*r*^2^ = 0.01, *P* = 0.2). To gain a further understanding of the connection between host genetic and gut microbiota, the correlations between host IBS matrix and Bray-Curtis distance were calculated, and the average correlation was 0.06 (*P* = 0.028), which may suggest that the composition of the microbial community is more dependent on the specific genotype of the individual rather than the genetic structure of the population. However, the influence of genetic factors on domestic ducks may be limited and influenced by additional factors. Furthermore, we found that the genetic diversity index of the host showed a significant correlation with microbial richness index (heterozygote rate: *R*^2^ = 0.18, *P* = 0.01; Homozygosity coefficient: *R*^2^ = −0.18, *P* = 0.01; Supplementary Figure S3A), while the association with the Shannon index approached suggested significance (heterozygote rate: *R*^2^ = 0.13, *P* = 0.06; Homozygosity coefficient: *R*^2^ = −0.13, *P* = 0.06; Supplementary Figure S3B). These results suggest that the genetic diversity of the host has a significant impact on the species richness of the microbial community, but has little effect on the distribution of microbiota.

### Core gut microbiota and breed-specific biomarkers in ducks

Among the 15 Chinese local duck breeds, 47 core genera were found in more than 95% of the individuals, which belonged to six phyla (Supplementary Figure S4A; Supplementary Table S4). In a single breed, we classified the genera with relative abundance > 0.1% as the core microbiome of this breed, and a total of 141 genera were detected in 15 duck breeds (Supplementary Table S5). SXP contained the most core genera (78), while PTW contained the fewest (25). We found that the abundance of 10 genera in each duck breed was > 0.1% abundance, including *Bacteroides*, *Corynebacterium*, *Enterococcus*, *Erysipelatoclostridium*, *Faecalitalea*, *Jeotgalicoccus*, *Lactobacillus*, *Streptococcus*, *Subdoligranulum*, and *UCG-005*.

**FIGURE 3:**
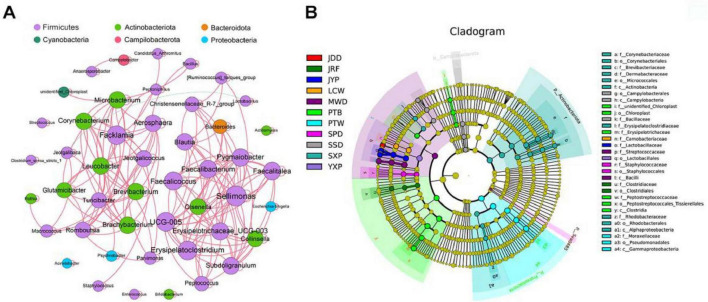
The correlations among core microbiomes and the cladogram of breed-specific biomarkers. **(A)** Co-occurrence networks of the core bacterial genera. A connection represents a strong correlation (Spearman’s correlation coefficient *r* > 0.6) and significant (*P* < 0.01). The size of each node is proportional to the number of connections (degree) and line color reflects direction (green: negative, red: positive). **(B)** Cladogram representation of the breed-specific biomarkers (see Supplementary Table S7). The root of the cladogram denotes the domain bacteria. The phyla are labeled, while class, order, and family are abbreviated, with the colors indicating the breed/line hosting the greatest abundance. The size of each node represents their relative abundance.

A network was constructed to reveal the significant correlations among 47 core genera in ducks (Spearman’s *r* > 0.6, *P* < 0.05). Most of the genera were positively significantly correlated with each other (Figure 3A; Supplementary Table S6). From the results, we observed that the network was divided into two major modules. The first module was mainly composed of Firmicutes, and the second module was composed of Firmicutes and Actinobacteriota. The most densely connected nodes in two modules were *Sellimonas* and *Facklamia*, which were defined as indicators for co-occurrence among core genera.

Linear discriminant analysis Effect Size (LEfSe) analysis was performed to identify species taxa with significantly different abundance among different groups and acted as biomarkers. In total, We identified a total of 57 biomarkers when the LDA score > 4, including four phyla, Actinobacteriota (SXP), Campilobacterota (SSD), Sva0485 (SPD), and Proteobacteria (PTW); and 12 genera were identified in eight breeds (Figure 3B; Supplementary Figure S4B; Supplementary Table S7), which SXP had the greatest number of breed-specific biomarkers (4).

### Prediction of gut microbial function in different duck breeds

**FIGURE 4:**
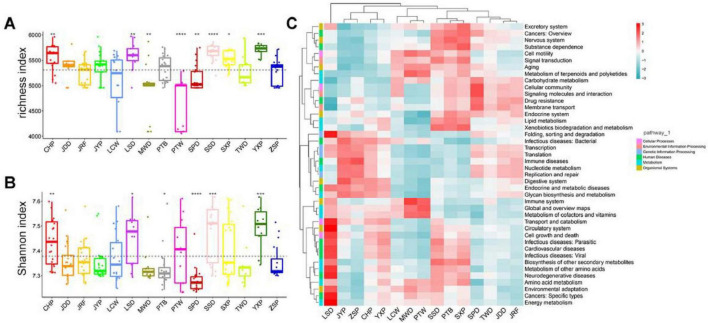
Comparison of gut bacterial Functions among duck breeds. **(A,B)** Alpha diversity (richness index) and Shannon indices of 15 duck breeds, respectively. Boxes denote the interquartile (IQR) between the first and third quartiles (25th and 75th percentiles, respectively), and the line inside denotes the median. The asterisks on the top indicate *P < 0.05, **P < 0.01, ***P < 0.001, and ****P < 0.0001 (Wilcoxon test). **(C)**. Bacterial Functions cluster analysis by heatmap.

The gut microbiome in different duck breeds was further analyzed with Tax4Fun to predict their potential function. Kyoto Encyclopedia of Genes and Genomes (KEGG) annotation revealed that 6,001 KEGG ortholog (KO) functions were identified in total (Supplementary Table S8). The number of KO in these samples ranged from 4,055 to 5,971, of which 3,971 KOs were distributed in all individuals as core functions in the gut microbiome of ducks. These KO functions were mapped into 42 KEGG level 2 categories (Supplementary Table S9). Most functions belonged to metabolism (79.9%), including amino acid metabolism (7.03%), carbohydrate metabolism (11.73%), energy metabolism (3.89%), and nucleotide metabolism (4.55%) (Supplementary Figure S5A). We further compared alpha diversity (richness and Shannon indices) in the individuals at the KO level (Figures 4A,B) and found that the richness and Shannon indices in CHP, LSD, SSD, and the Youxian Partridge Duck (YXP) were significantly higher than the average of the population, while these indices were significantly lower in SPD.

Partitioning of beta diversity indicated that the gut microbial functions of most duck breeds had similar distances on the PC1, which accounted for 57.48% of the observed variation, indicating that these functions in most duck breeds were similar. However, PC2, representing 23.22% of the variation, was associated with the different breeds (Supplementary Figure S5B). ANOSIM analysis (*r* = 0.41, *P* = 0.001) also highlighted significant differences between chicken breeds (Supplementary Figure S5C). The clustering of 42 secondary pathways in different duck breeds showed that SSD, PTB, and SXP possessed similar, rich secondary pathways, and LSD was the most enriched in human diseases (Figure 4C). The relationship between the core gut microbiota and secondary KEGG pathways was analyzed using a correlation heatmap (Spearman’s *r* > 0.6, *P* < 0.01; Supplementary Figure S5D; Supplementary Table S10). *Lactobacillus* was strongly positively correlated with glycan biosynthesis and metabolism, digestive system, nucleotide metabolism, and strongly negatively correlated with metabolism of terpenoids and polyketides, and aging, while numerous bacteria, such as *Jeotgalibaca* and *Aerosphaera*, showed the opposite tendency in most functions.

### Identification of enterotype and its functions in ducks

**FIGURE 5:**
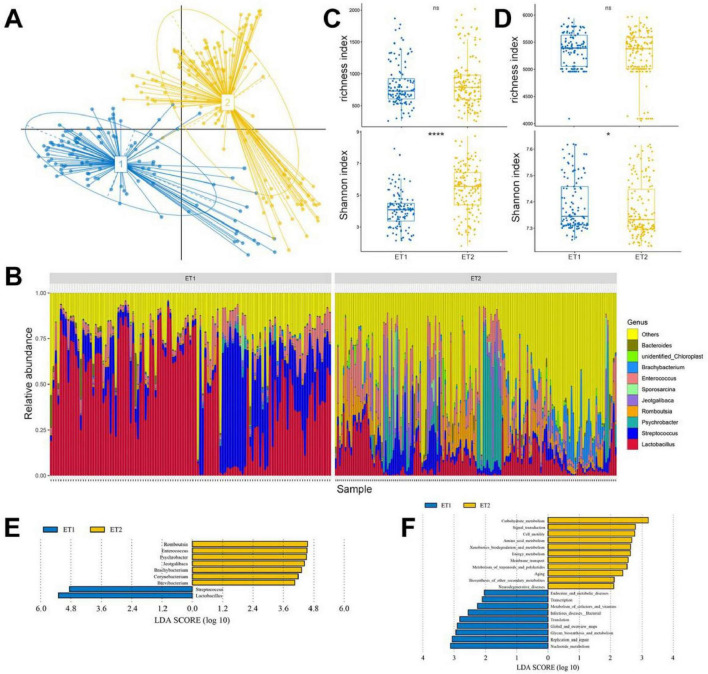
Enterotype distributions of duck gut microbiota using Jensen–Shannon divergence dissimilarity. **(A)** Visualizations of enterotypes, as identified by PAM (partitioning around medoid) clustering. **(B)** Alpha diversity of the gut microbiota compositions. **(C)** Alpha diversity of the gut microbiota functions. **(D)** The relative abundance of the 10 most abundant genera. **(E)** LEfSe analysis shows differentially abundant genera as biomarkers determined using Kruskal–Wallis test (*P* < 0.05) with LDA score > 4. **(F)** Lefse analysis of KEGG level 2 in two enterotypes (LDA score > 2).

We calculated the Jensen–Shannon distance (JSD) based on the relative abundances of bacteria at the genus level, and the highest Calinski–Harabasz (CH) index value was obtained for two clusters (Figure 5A; Supplementary Figures S6A,B). The analysis was repeated with the Bray–Curtis (BC) dissimilarity and found that two clusters were the most desirable as well (Supplementary Figures S6C,D). The JSD PCoA was used to divide and display two enterotypes. Enterotype 1 (ET1) was mostly composed of CHP, the Ji’an red Duck (JRF), JYP, TWD, MWD, YXP, and the Zhongshan Partridge Duck (ZSP), whereas enterotype 2 (ET2) was majorly composed of the Jingding Duck (JDD), LCW, PTW, PTB, SPD, SSD, and SXP (Supplementary Table S11), and the gut microbiota of LSD was equally distributed in two enterotypes. According to SIMPER analysis, each cluster was driven by the variation of the abundance of the representative genera: *Lactobacillus* and *Streptococcus* in ET1, *Psychrobacter*, *Romboutsia*, and *Jeotgalibaca* in ET2 (Table 1). Genera corresponding to each enterotype were identified by their relative abundance (Figure 5B).

**TABLE 1:** The result of the similarity percentage (SIMPER) analysis among two enterotypes.

Genus	Average abundance		Contribution (%)
	ET1 (%)	ET2 (%)	
*Lactobacillus*	46.72	9.00	26.56
*Streptococcus*	20.81	6.78	11.51
*Psychrobacter*	0.54	6.85	7.97
*Romboutsia*	1.05	6.75	7.44
*Jeotgalibaca*	0.51	5.33	5.53
*Sporosarcina*	0.24	0.84	4.89
*Enterococcus*	6.54	11.7	4.25
*Brachybacterium*	0.40	3.86	2.89
*unidentified_Chloroplast*	0.12	1.28	2.10
*Bacteroides*	1.20	1.28	1.64

The composition and functions of ET1 and ET2 were first assessed using richness and Shannon indices. We found that two enterotypes shared similar species composition and richness index, and ET2 presented higher Shannon index values than ET1 (*P* < 0.001; Figure 5C). As with the species compositions of ET1 and ET2, the functions shared similar richness and different Shannon indices, but ET1 had higher Shannon index values than ET2, indicating that although ET2 contained more diverse bacteria, the functions of these microbiota were simpler than those in ET1 (Figure 5D). To identify the feature species and functions in ET1 and ET2, we conducted LEfSe analysis. Species enriched in ET1 were mainly from the genera *Lactobacillus* and *Streptococcus* (LDA > 4). Species enriched in ET2 were mainly from the genera *Romboutsia*, *Enterococcus*, *Psychrobacter, Jeotgalibaca*, *Brachybacterium*, *Corynebacterium*, and *Brevibacterium* (Figure 5E). Regarding molecular function, ET1 was enriched with genes involved in nucleotide metabolism, replication and repair, glycan biosynthesis, and metabolism, and ET2 was enriched with those involved in carbohydrate, amino acid, and energy metabolism (Figure 5F).

Co-occurrence network of the top 20 genera revealed the correlation of these gut microbiota in two enterotypes (Spearman’s *r* > 0.6, *P* < 0.01; Supplementary Figure S6E; Supplementary Table S12). In ET1, positive correlations were found among the members of Actinobacteriota, and the members of Firmicutes and Bacteroidota existed in unison. The most densely connected node was *UCG-005*, while *Lactobacillus*, the most abundant genus, was negatively associated with most of these genera. The network complexity in ET2 (edge: 41, modularity: 0.619, average degree: 4.1) was higher than ET1 (edge: 36, modularity: 0.407, average degree: 3.6). The largest co-occurrence network consisted of the members of Firmicutes, Actinobacteriota, and Proteobacteria, and the abundance of *Romboutsia* had a significant correlation with the abundance of most genera.

## Discussion

In the past decade, many studies in gut microbiota of domestic ducks have focused on a single breed (Wang et al., 2018; Zhu et al., 2020). However, few studies have compared the gut microbiomes of different duck breeds. To the best of our knowledge this is the first large-scale study exploring the relationship between different duck breeds and gut microbiota composition. The number of indigenous duck breeds involved in this study was almost half the number of the total local duck breeds in China, and seven breeds are now protected breeds of livestock and poultry genetic resources, thus, these experimental breeds were well represented in this study. In addition, all duck breeds involved in this experiment were born and raised in the same farm environment to eliminate variables (differences in diet and environment) that may influence host genetics on gut microbiota (Trevelline et al., 2020). Furthermore, adult individuals have stable and mature microbial communities (Schreuder et al., 2019), thus, we collected fresh feces from 260 adult ducks (120 day-old) for the analysis of gut microbiome.

The alpha diversity indices demonstrated that the gut microbiota of SSD and SXP was more abundant and diverse than that in other breeds, while these indices were significantly lower in LCW, MWD and PTW. And in some cases, the genetic diversity and the alpha diversity showed a remarkable degree of agreemant. Hosts with high genetic diversity may possess enhanced adaptive capabilities, thereby supporting a greater variety of microbial communities. And the rich and diverse gut microbiota was the embodiment of intestinal health in the host, while low diversity of gut microbiome was a good predictor of poor health status (Heiman and Greenway, 2016; Le Chatelier et al., 2013). The association between the host genetic kinship and microbial Bray-Curtis distance showed that each duck had a unique gut microbial structure, and individuals of the same breed had more similar gut microbial structures compared to different breeds, which suggested that the composition and structure of gut microbiota could be correlated with genetic background. Many scholars have shown that host heredity plays an important role in gut microbiota. For example, Deschasaux et al. (2018) explored the differences of the gut microbial composition in a population of various ethnic origins but shared geography, and found that people which have the same ethnic background and live in the same city tend to have similar gut microbial structures, and ethnicity contributed to interindividual dissimilarities in gut microbiota composition. Bergamaschi et al. (2020) characterized differences in the fecal microbial composition of three commercially relevant pig breeds living on the same farm and suggested that host genetics had an essential effect on the structure and composition of pig gut microbiome. In addition, Pandit et al. (2018) found that although chickens were raised in different geographical locations, different breeds or strains of chickens were still clustered into different clusters in PCoA, indicating a host component in microbiome composition. In this experiment, external factors such as environment and diet were controlled, allowing us to observed the effects of breed difference on gut microbiota of domestic ducks, suggested that the breed specificity was associated with distinct gut microbial compositions. Certainly, the correlation between gut microbiota composition and host genetic variation was relatively weak, which is consistent with previous studies (Trevelline et al., 2020). other intrinsic factors may also contribute to the variation in gut microbiota, such as epigenetic modifications (Ferenc et al., 2024; Louwies et al., 2020), maternal microbial transmission (Chen et al., 2020; Ferretti et al., 2018), and unmeasured environmental exposures. Further research incorporating more comprehensive host and environmental data are needed to more accurately disentangle the contribution of host genetics to the structure of the gut microbiota.

When comparing host species, we found that the duck gut microbiota showed great similarities at the phylum level, while the gut microbial composition differed according to species at the genus level. Regarding these similarities, we found that the gut microbiomes of duck breeds were dominated by the phyla Firmicutes, Proteobacteria, Actinobacteriota, and Bacteroidota, suggesting that the dominant phyla in domestic ducks gut were similar to those in other poultry (Waite and Taylor, 2015; Xiao et al., 2017) and wild birds (Cao et al., 2020; Gao et al., 2021). Firmicutes was dominant in all duck breeds, and its members might promote host metabolism and digestion through their ability to produce short-chain fatty acids through the breakdown of dietary carbohydrates and polysaccharides (Trevelline et al., 2020). Bacteroidota ranked fourth in abundance in the duck’s gut. Previous studies have shown that higher Firmicutes/Bacteroidota ratios are associated with human obesity, and the reverse has been linked with weight loss (Koliada et al., 2017). However, whether a similar pattern is followed in ducks requires further investigation. In Addition, we found that PTW ducks harbored a higher abundance of *Proteobacteria* and significantly lower alpha diversity compared to other breeds. It is well known that an elevated level of *Proteobacteria* is often considered a potential marker of gut dysbiosis (Shin et al., 2015), while low alpha diversity typically reflects a simplified microbial community structure with reduced resilience, which may indicate a less stable gut microecosystem (Videvall et al., 2020). At the genus level, the relative abundance of the dominant bacteria varied significantly at different duck breeds. *Lactobacillus* was the most abundant genus in most domestic ducks, especially in JYP, and is generally considered to be a probiotic; it produces vitamins and organic acids, competitively inhibits pathogens (Martin et al., 2013; Neal-McKinney et al., 2012). The dual-purpose type ducks, including CHP, JRF, LSD, and ZSP, were usually dominated by *Lactobacillus*. It is possible that *Lactobacillus* improves meat quality (Wang et al., 2017). Other high-abundance genera such as *Streptococcus*, *Psychrobacter*, *Romboutsia*, and *Brachybacterium* were found in normal gut microflora that hydrolyzed starch or produced short chain fatty acids, and are involved in immunity and gut barrier protection (Andam and Hanage, 2015; Huang et al., 2018; Mangifesta et al., 2018; Wescombe et al., 2009). *Romboutsia* is known to enhance the host’s immune function and improve the overall composition of the gut microbiota (Song et al., 2024). *Streptococcus* is a normal part of the gut microbiota, and certain species within this genus play a key role in regulating host intestinal health, such as *Streptococcus thermophilus* (Mirsalami and Mirsalami, 2024) and *Streptococcus salivarius* (Zhang et al., 2007). *Brachybacterium* was widely present in poultry farming environments, fluctuations in its abundance may affect the host’s production performance (Bindari et al., 2021). These genera, as distinct LEfSe biomarkers also defined different duck breeds or lines, which might suggest the host genetics background contributed to the differences in gut microbial structure in domestic ducks.

In this study, we identified a total of 47 core genera, most of which were members of Firmicutes and Actinobacteriota, with strong positive correlations between their members, indicating that they may have a consistent response to similar environmental conditions (Ning et al., 2020). *Sellimonas* and *Facklamia* were the keystone members of 47 core gut bacterial co-occurrence network among domestic ducks, which play important roles in pathogenic bacteria resistance and maintaining gut microbial homeostasis (Liang et al., 2020; Munoz et al., 2020). In addition, the zoonotic pathogens *Enterococcus*, *Helicobacter*, *Campylobacter*, and *Fusobacterium* were also detected at abundances > 0.1% in several duck breeds, especially in LSD, which might contribute to the enrichment in KEGG level 1 pathway human diseases. In addition, these pathogens would affect the gut microenvironment and cause inflammatory immune responses (Pandit et al., 2018). Further study is required to determine the relative contributions of the host and these pathogens to the differences in gut microbiota.

The clustering of enterotypes was not affected by factors such as age, gender, and geographical location, and can reflect the changes of ecological niches at the genus level (Costea et al., 2018). The representative breeds of the two enterotypes were different, among which the representative breeds of ET1 were dual-purpose type, while those of the ET2 were layer ducks. In chicken, the gut microbiota is more complex and richer in layers than in broilers (Khan et al., 2020), which is in agreement with our findings. In KEGG functions, the genes of nucleotide metabolism, replication and repair, and glycan biosynthesis and metabolism were enriched in ET1. These enriched functions may contribute to enhanced host growth performance by promoting glycogen accumulation and fat synthesis (Cui et al., 2021), which aligns with the functional needs of dual-purpose duck breeds predominantly represented in ET1 (Ouyang et al., 2023). And ET2 was enriched with genes involved in Some KEGG level 2 pathways, including carbohydrate, amino acid, and energy metabolism, which showed that the enterotype was more inclined toward energy utilization, and the enterotype with a significant enrichment of KOs in carbohydrate metabolism pathways had a better digestive capacity for cereal-based diets (Ramayo-Caldas et al., 2016), and might provide the host with greater energy availability, thereby supporting sustained high levels of egg production (Yang et al., 2022). However, functional prediction using Tax4Fun based on 16S rRNA data are inherently limited. Future research incorporating multi-omics approaches will be essential for a more comprehensive and accurate characterization of the duck microbial function.

## Conclusion

The effect of different duck breeds (with the same diet and environment) on gut microbiome was discovered using 16 S rRNA gene amplicon sequencing and WGS analysis. We found that individuals of the same breed tended to have similar gut microbiota compared to that of different breeds, which suggested that differences in gut microbiota among breeds were partially influenced by genetic variaions. The annotated functions showed similar trends among the gut bacterial communities of different duck breeds. The JSD PcoA analysis showed that the 260 samples formed two distinct enterotype clusters, with representative breeds corresponding to layer ducks and dual-purpose ducks, respectively. Each cluster was driven by the variation in abundance of the representative genera, rather than the presence or absence of specific species, leading to the enrichment of distinct functional profiles. Our study provides a comprehensive biological insight into the gut microbiota of domestic ducks and reveals the influence of genetic background on gut microbial composition.

## Data Availability

The raw sequencing data of 16s rDNA from the 260 fecal samples is available from Genome Sequence Archive (GSA) database under accession number PRJCA015941.

## Footnotes

1
http://github.com/samtools/samtools

2
https://software.broadinstitute.org/gatk/

## Supplementary material

Supplementary Figure S1 The quality of sequencing results and the distribution of operational taxonomic units (OTUs). **(A)** Petal map based on OTUs. The middle core number represents the common OTUs of all breeds, and the number on the petals represents the unique OTU number of each breed. **(B)** Species accumulation boxplot. **(C)** Rarefaction curves of OTUs at 97% similarity for each sample. **(D)** Rank abundance curves. The *x*-coordinate is the sorting number and the *y*-coordinate is the relative abundance in OTUs.

Supplementary Figure S2 Comparison of gut microbiota composition among duck breeds. **(A)** UPGMA-clustering tree based on Bray–Curtis distance at the phylum level. **(B)** Box plot indicating differences in the ranked distance in each group (ANOSIM analysis). **(C,D)** represent the relative abundance of the top 10 phyla and genera in all breeds.

Supplementary Figure S3 Correlation between alpha diversity and genetic diversity. **(A)** The correlation between microbial richness index and genetic diversity index. **(B)** The correlation between microbial Shannon index and genetic diversity index.

Supplementary Figure S4 The core microbiomes and breed-specific biomarkers. **(A)** The core bacterial community cluster analysis by heatmap (see Supplementary Table S4). **(B)** LEfSe analysis based on characterizing discriminative feature of OTUs (LDA score > 4).

Supplementary Figure S5 The predictive KEGG functions in each group based on Tax4fun. **(A)** The relative abundance of secondary KEGG pathways. **(B)** PCoA plots based on Bray–Curtis distance. **(C)** Box plot revealed differences in the ranked distance in each group (ANOSIM analysis). **(D)** Heatmap of correlation between core microbiome and secondary KEGG pathways. Spearman’s correlation coefficient *r* > 0.6. The asterisk in the square **P* < 0.05, ***P* < 0.01, ****P* < 0.001 (see Supplementary Table S10).

Supplementary Figure S6 The identification of enterotype of domestic ducks. **(A,C)** Represented optimal number of clusters for partitioning around medoid (PAM) analysis based on Jensen–Shannon distance (JSD) and Bray–Curtis (BC) among the relative abundance distributions at the genus level. PCoA plots represent the two enterotype clusters of domestic duck’s gut microbiota identified using PAM based on the JSD **(B)** and BC **(D)** among relative abundance at the genus level. **(E)** Co-occurrence networks of the top 20 genera in the two enterotype (see Supplementary Table S12). Spearman’s correlation > 0.6 or < –0.6 is illustrated and line color reflects direction (green: negative; red: positive). The size of the node is proportional to the number of connections.
