# Supplementary figures and images for: Breed-specific gut microbiota and enterotype divergence in Chinese indigenous ducks
Source: Front Microbiol. 2025 Jul 17;16:1602641. doi: 10.3389/fmicb.2025.1602641 (PMC12312609; doi:10.3389/fmicb.2025.1602641)

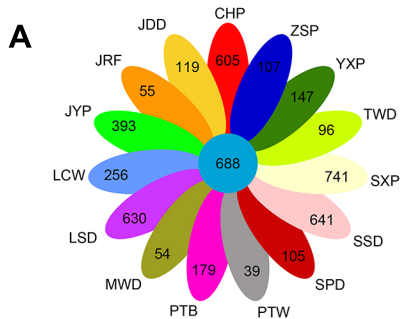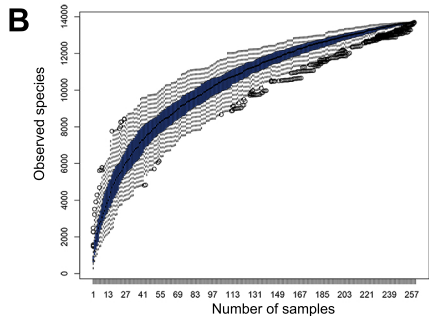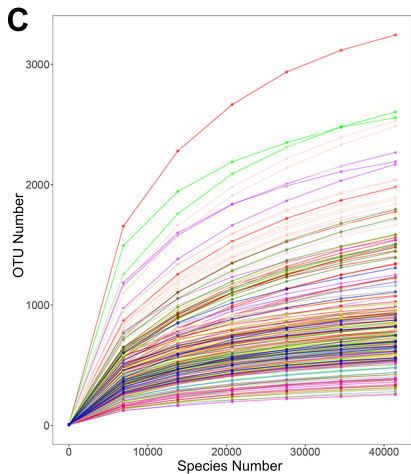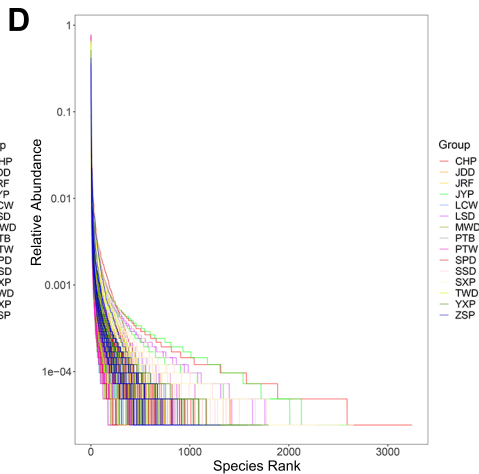

Supplement: Supplementary Figure S1 — The quality of sequencing results and the distribution of operational taxonomic units (OTUs). (A) Petal map based on OTUs. The middle core number represents the common OTUs of all breeds, and the number on the petals represents the unique OTU number of each breed. (B) Species accumulation boxplot. (C) Rarefaction curves of OTUs at 97% similarity for each sample. (D) Rank abundance curves. The x-coordinate is the sorting number and the y-coordinate is the relative abundance in OTUs. [file Data_Sheet_1.pdf]

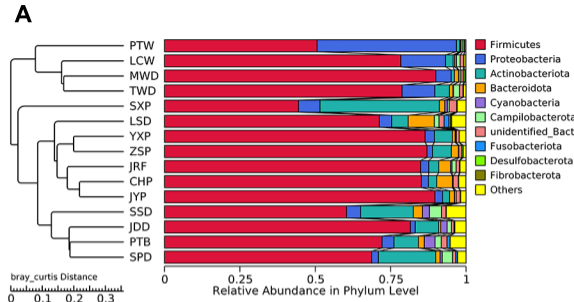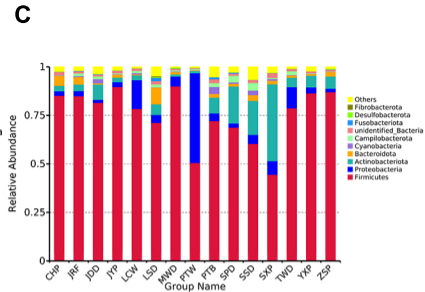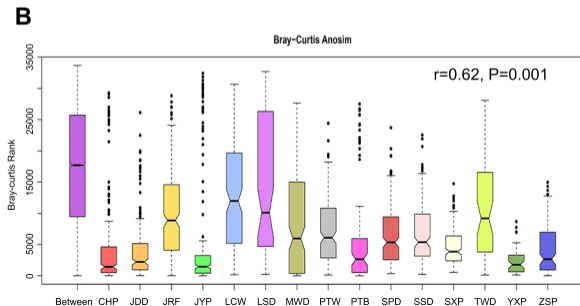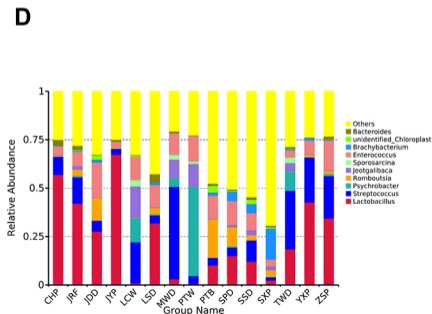

Supplement: Supplementary Figure S2 — Comparison of gut microbiota composition among duck breeds. (A) UPGMA-clustering tree based on Bray–Curtis distance at the phylum level. (B) Box plot indicating differences in the ranked distance in each group (ANOSIM analysis). (C,D) represent the relative abundance of the top 10 phyla and genera in all breeds. [file Data_Sheet_2.pdf]

**A**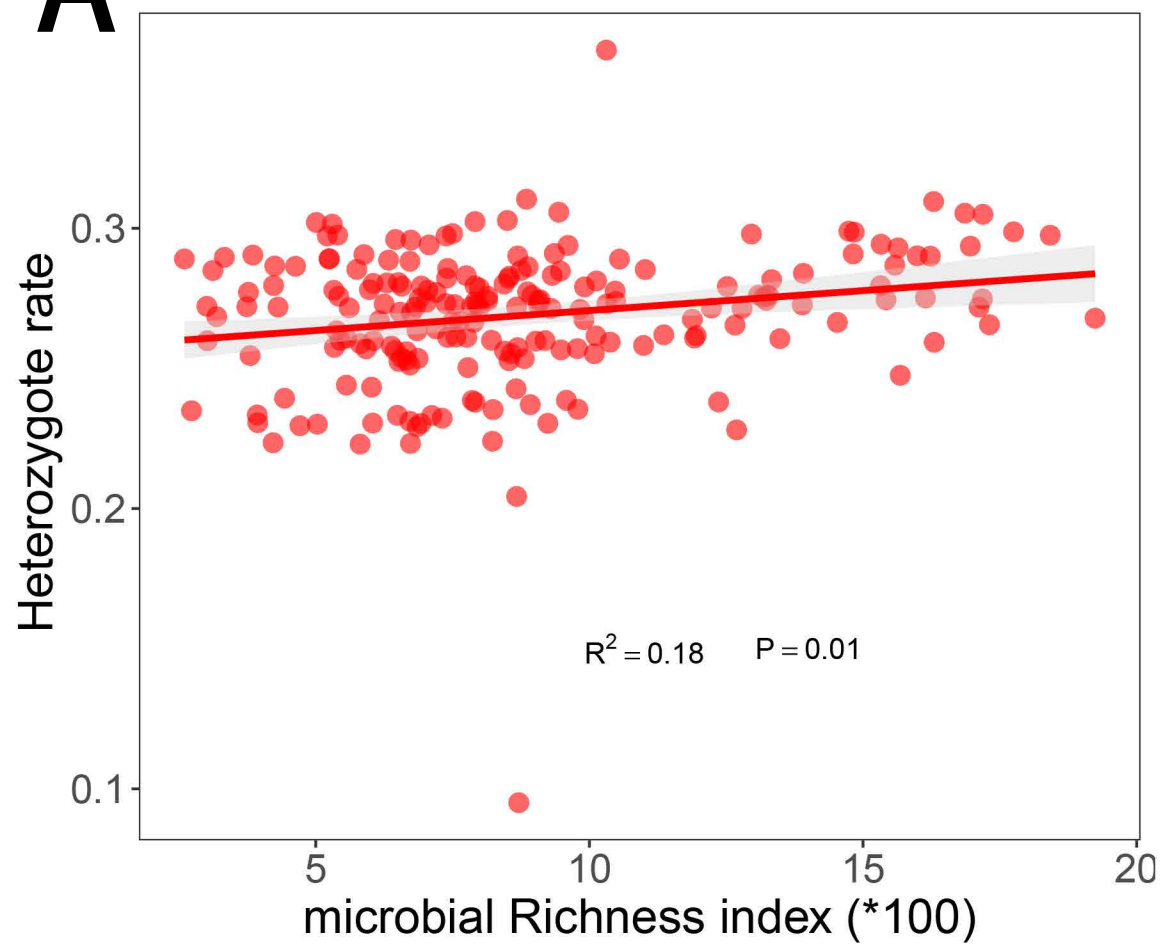**B**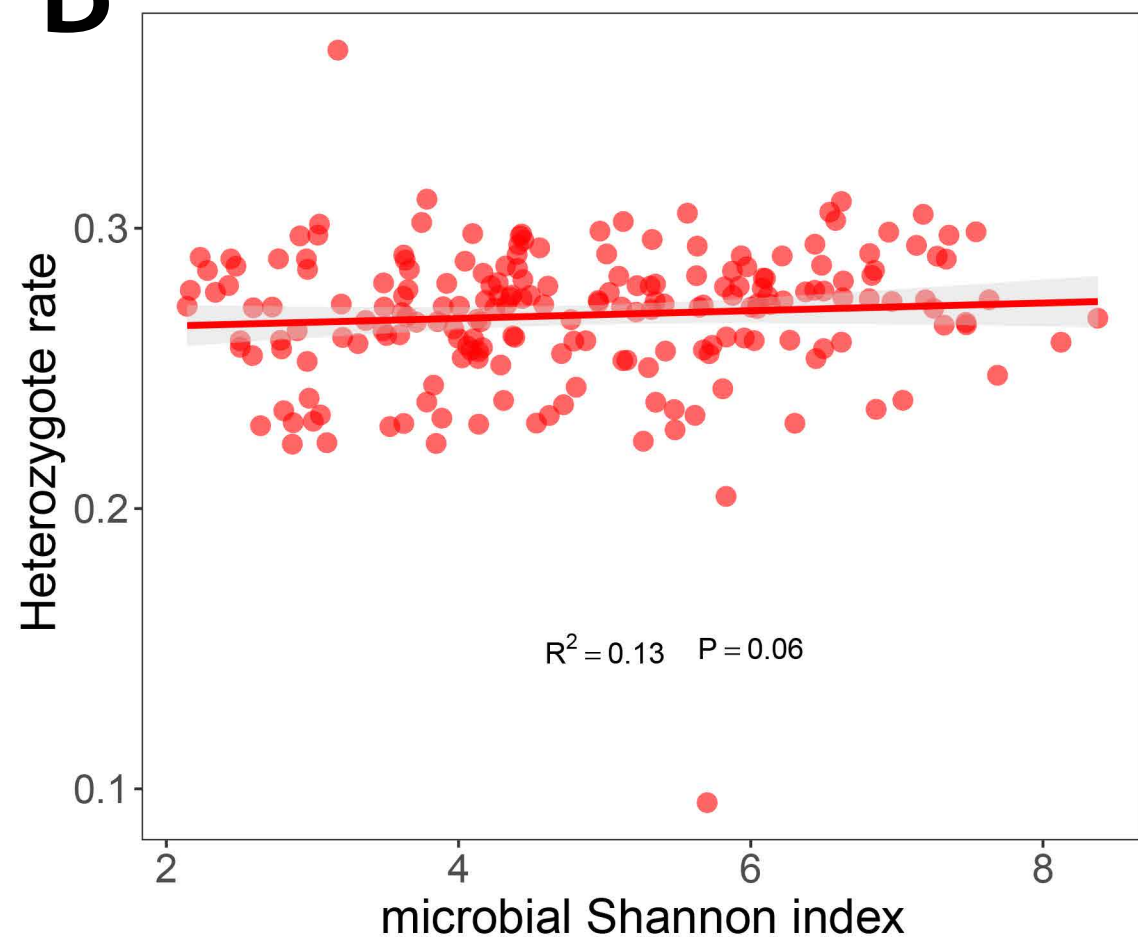**Homozygosity coefficient**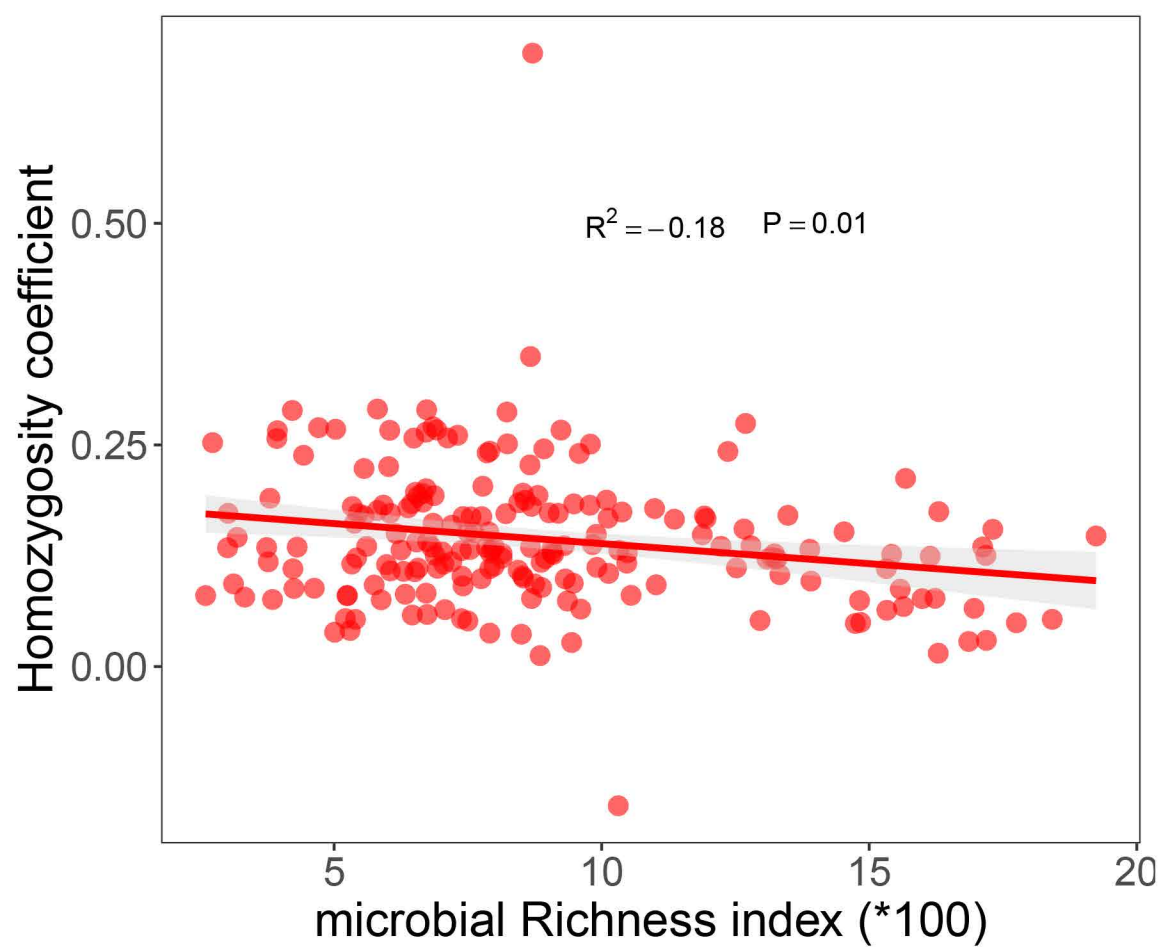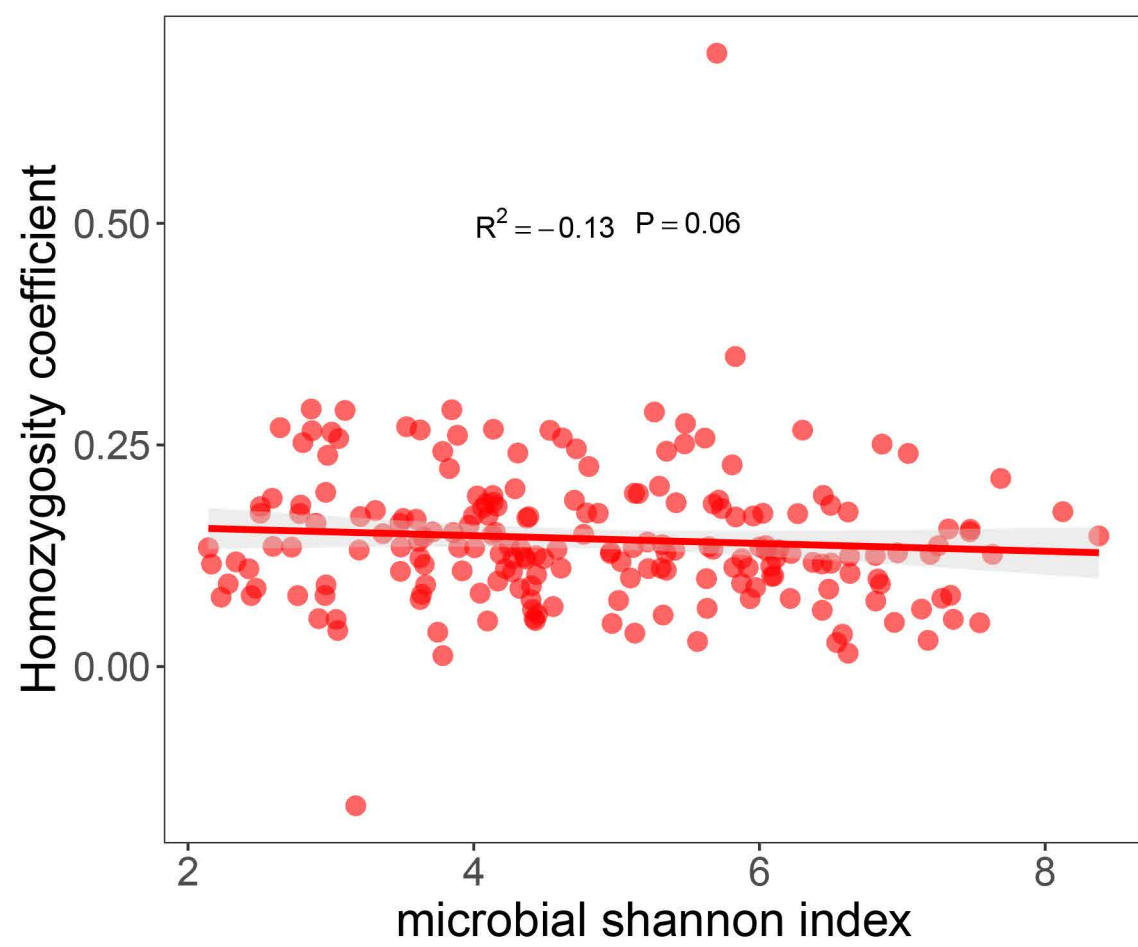

Supplement: Supplementary Figure S3 — Correlation between alpha diversity and genetic diversity. (A) The correlation between microbial richness index and genetic diversity index. (B) The correlation between microbial Shannon index and genetic diversity index. [file Data_Sheet_3.pdf]

A

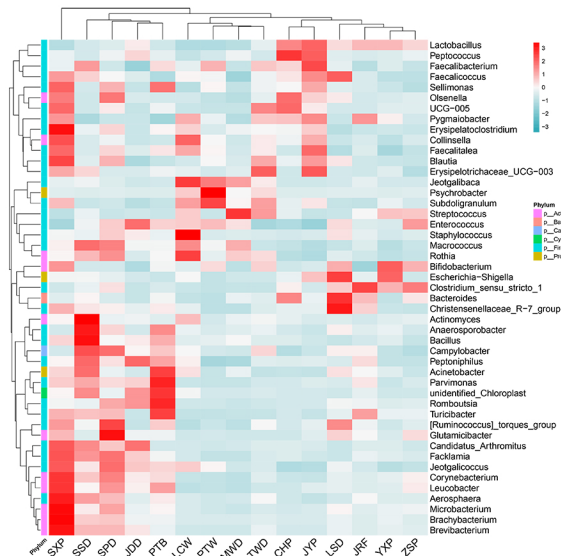

B

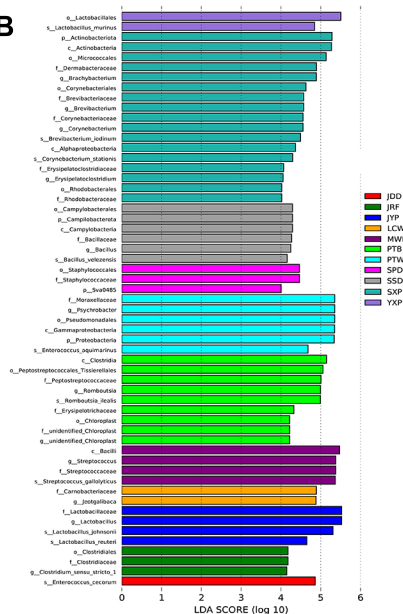

Supplement: Supplementary Figure S4 — The core microbiomes and breed-specific biomarkers. (A) The core bacterial community cluster analysis by heatmap (see Supplementary Table S4). (B) LEfSe analysis based on characterizing discriminative feature of OTUs (LDA score > 4). [file Data_Sheet_4.pdf]

**A**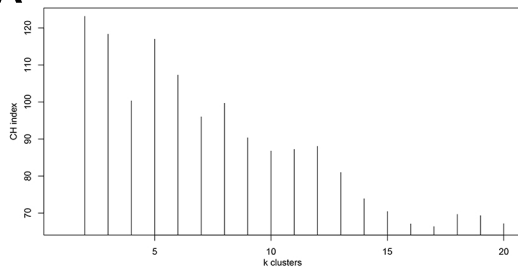**B**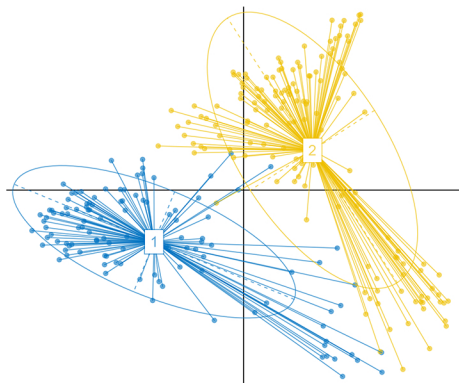**C**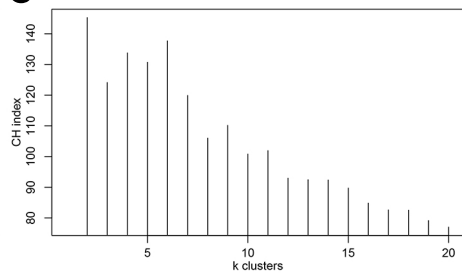**D**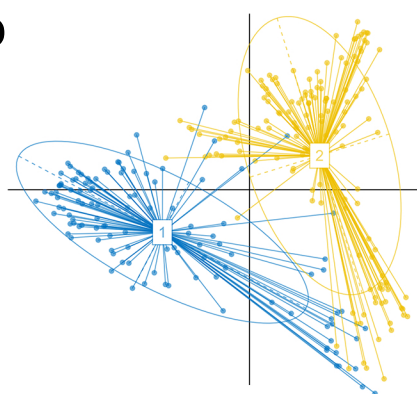**E**

ET1

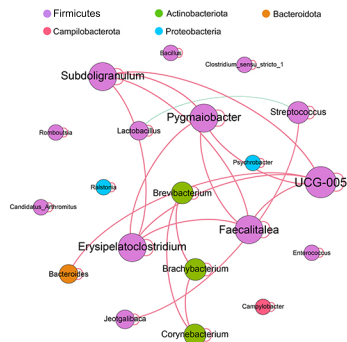

ET2

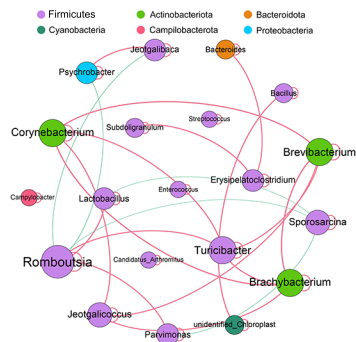

Supplement: Supplementary Figure S6 — The identification of enterotype of domestic ducks. (A,C) Represented optimal number of clusters for partitioning around medoid (PAM) analysis based on Jensen–Shannon distance (JSD) and Bray–Curtis (BC) among the relative abundance distributions at the genus level. PCoA plots represent the two enterotype clusters of domestic duck’s gut microbiota identified using PAM based on the JSD (B) and BC (D) among relative abundance at the genus level. (E) Co-occurrence networks of the top 20 genera in the two enterotype (see Supplementary Table S12). Spearman’s correlation > 0.6 or < –0.6 is illustrated and line color reflects direction (green: negative; red: positive). The size of the node is proportional to the number of connections. [file Data_Sheet_6.pdf]
