# Supplementary material for: Breed-specific gut microbiota and enterotype divergence in Chinese indigenous ducks
Source: Front Microbiol. 2025 Jul 17;16:1602641. doi: 10.3389/fmicb.2025.1602641 (PMC12312609; doi:10.3389/fmicb.2025.1602641)
Supplement: Supplementary Figure S5 — The predictive KEGG functions in each group based on Tax4fun. (A) The relative abundance of secondary KEGG pathways. (B) PCoA plots based on Bray–Curtis distance. (C) Box plot revealed differences in the ranked distance in each group (ANOSIM analysis). (D) Heatmap of correlation between core microbiome and secondary KEGG pathways. Spearman’s correlation coefficient r > 0.6. The asterisk in the square *P < 0.05, **P < 0.01, ***P < 0.001 (see Supplementary Table S10). [file Data_Sheet_5.pdf]

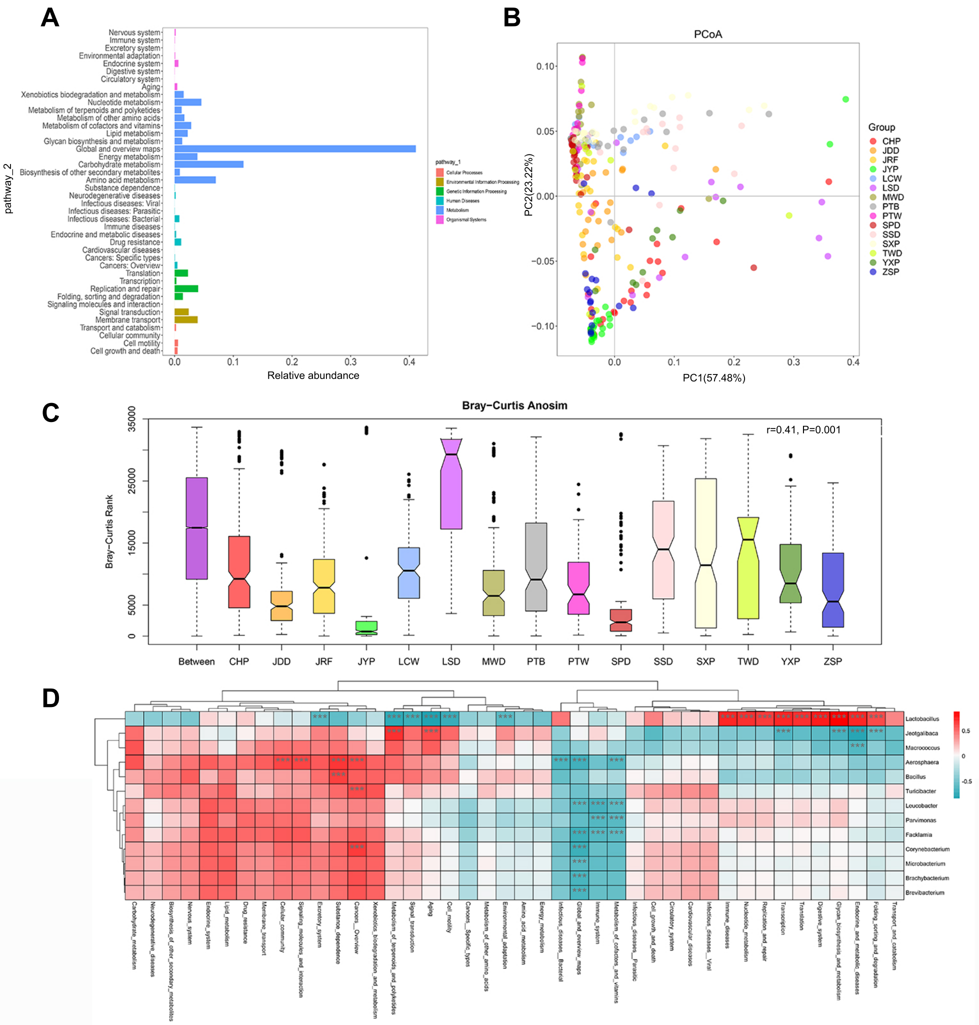

A
Nervous system
Immune system
Excretory system
Environmental adaptation
Endocrine system
Digestive system
Circulatory system
Aging
Xenobiotics biodegradation and metabolism
Nucleotide metabolism
Metabolism of terpenoids and polyketides
Metabolism of other amino acids
Metabolism of cofactors and vitamins
Lipid metabolism
Glycan biosynthesis and metabolism
Global and overview maps
Energy metabolism
Carbohydrate metabolism
Biosynthesis of other secondary metabolites
Amino acid metabolism
Substance dependence
Neurodegenerative diseases
Infectious diseases: Viral
Infectious diseases: Parasitic
Infectious diseases: Bacterial
Immune diseases
Endocrine and metabolic diseases
Drug resistance
Cardiovascular diseases
Cancers: Specific types
Cancers: Overview
Translation
Transcription
Replication and repair
Folding, sorting and degradation
Signaling molecules and interaction
Signal transduction
Membrane transport
Transport and catabolism
Cellular community
Cell motility
Cell growth and death
pathway_2
0.0
0.1
0.2
0.3
0.4
Relative abundance
pathway_1
Cellular Processes
Environmental Information Processing
Genetic Information Processing
Human Diseases
Metabolism
Organismal Systems
B
PCoA
0.10
0.05
0.00
−0.05
−0.10
PC2(23.22%)
0.0
0.1
0.2
0.3
0.4
PC1(57.48%)
Group
CHP
JDD
JRF
JYP
LCW
LSD
MWD
PTB
PTW
SPD
SSD
SXP
TWD
YXP
ZSP
C
Bray−Curtis Anosim
r=0.41, P=0.001
Bray-Curtis Rank
35000
25000
15000
5000
0
Between
CHP
JDD
JRF
JYP
LCW
LSD
MWD
PTB
PTW
SPD
SSD
SXP
TWD
YXP
ZSP
D
Lactobacillus
Jeotgalibaca
Macrococcus
Aerosphaera
Bacillus
Turicibacter
Leucobacter
Parvimonas
Facklamia
Corynebacterium
Microbacterium
Brachybacterium
Brevibacterium
0.5
0
-0.5
Carbohydrate_metabolism
Neurodegenerative_diseases
Biosynthesis_of_other_secondary_metabolites
Nervous_system
Endocrine_system
Lipid_metabolism
Drug_resistance
Membrane_transport
Cellular_community
Signaling_molecules_and_interaction
Excretory_system
Substance_dependence
Cancers__Overview
Xenobiotics_biodegradation_and_metabolism
Metabolism_of_terpenoids_and_polyketides
Signal_transduction
Aging
Cell_motility
Cancers__Specific_types
Metabolism_of_other_amino_acids
Environmental_adaptation
Amino_acid_metabolism
Energy_metabolism
Infectious_diseases__Bacterial
Global_and_overview_maps
Immune_system
Metabolism_of_cofactors_and_vitamins
Infectious_diseases__Parasitic
Cell_growth_and_death
Circulatory_system
Cardiovascular_diseases
Infectious_diseases__Viral
Immune_diseases
Nucleotide_metabolism
Replication_and_repair
Transcription
Translation
Digestive_system
Glycan_biosynthesis_and_metabolism
Endocrine_and_metabolic_diseases
Folding_sorting_and_degradation
Transport_and_catabolism
